# Lap Shear Strength and Fatigue Analysis of Continuous Carbon-Fibre-Reinforced 3D-Printed Thermoplastic Composites by Varying the Load and Fibre Content

**DOI:** 10.3390/polym16050579

**Published:** 2024-02-21

**Authors:** Khalid Saeed, Alistair Mcilhagger, Thomas Dooher, Jawad Ullah, Faisal Manzoor, Xavier Velay, Edward Archer

**Affiliations:** 1 Mechanical Engineering Department, Atlantic Technological University, Ash Lane Sligo, F91 YW50 County Sligo, Ireland; xavier.velay@atu.ie; 2 Engineering Research Institute, Ulster University, York Street, Belfast BT1 5ED, Co. Antrim, UK; a.mcilhagger@ulster.ac.uk (A.M.); j.ullah@ulster.ac.uk (J.U.); f.manzoor@ulster.ac.uk (F.M.); 3 The Northern Ireland Advanced Composites and Engineering Centre, Belfast BT3 9EF, Co. Antrim, UK; t.dooher@niace.org

**Keywords:** additive manufacturing, lap shear strength, fatigue analysis, 3D printing, cyclic loading, stress ratio

## Abstract

This study focuses on evaluating the fatigue life performance of 3D-printed polymer composites produced through the fused deposition modelling (FDM) technique. Fatigue life assessment is essential in designing components for industries like aerospace, medical, and automotive, as it provides an estimate of the component’s safe service life during operation. While there is a lack of detailed research on the fatigue behaviour of 3D-printed polymer composites, this paper aims to fill that gap. Fatigue tests were conducted on the 3D-printed polymer composites under various loading conditions, and static (tensile) tests were performed to determine their ultimate tensile strength. The fatigue testing load ranged from 80% to 98% of the total static load. The results showed that the fatigue life of the pressed samples using a platen press was significantly better than that of the non-pressed samples. Samples subjected to fatigue testing at 80% of the ultimate tensile strength (UTS) did not experience failure even after 1 million cycles, while samples tested at 90% of UTS failed after 50,000 cycles, with the failure being characterized as splitting and clamp area failure. This study also included a lap shear analysis of the 3D-printed samples, comparing those that were bonded using a two-part Araldite glue to those that were fabricated as a single piece using the Markforged Mark Two 3D printer. In summary, this study sheds light on the fatigue life performance of 3D-printed polymer composites fabricated using the FDM technique. The results suggest that the use of post-printing platen press improved the fatigue life of 3D-printed samples, and that single printed samples have better strength of about 265 MPa than adhesively bonded samples in which the strength was 56 MPa.

## 1. Introduction

Additive manufacturing/3D printing is an advanced technology that is used to produce intricate parts at low cost and higher accuracy. Three-dimensional printing offers several benefits, such as rapid transition from design to prototype, and the ability to produce intricate parts with minimum material wastage in comparison to traditional manufacturing methods. Fused deposition modelling (FDM) is one of the promising AM techniques widely used to manufacture complex and intricate structures using material in filament form. Polymer composites are materials that are composed of a polymer matrix and a reinforcing material, resulting in a material that exhibits improved mechanical, thermal, and chemical characteristics. The polymer material provides flexibility, lightness, and ease of processing, while the reinforcing material contributes towards strength, stiffness, and durability. The reinforcing materials in polymer composites are in the form of fibre, such as carbon, glass, or Kevlar, or any other material. The combination of polymer matrix and reinforcing materials creates a synergistic effect, making the composite material suitable for a wide range of applications across various industries. Polymer composites are used in aerospace, automotive, construction, electronics, marine, and sports equipment.

Different types of failure are known to occur due to loading during the operation of structures. According to research, more than 50% of the failures are due to fatigue. Cyclic loading on materials leads to failure under fatigue. Therefore, it is important to know how a part would respond under recurring loading and to understand the durability and reliability of the part. Cyclic stress (fatigue) causes the failure of components at lower stress levels compared to static mechanical loading [1]. There are limited studies performed on the fatigue damage mechanism and fatigue life prediction methodologies for polymer composites. The fatigue damage in composites has four basic failure mechanisms: matrix cracking, delamination, fibre breakage, and interfacial debonding. Recently, Imeri et al. [2] studied the fatigue properties of continuous fibre-composite-reinforced specimens fabricated through additive manufacturing. Specimens with a stress ratio (S.R) of 0.1 were used for testing by varying the test parameters such as fibre positioning, infill type, and material compositions using a 3D printer which can reinforce with carbon fibre, glass fibre, and Kevlar. Tests were performed until the samples broke and completely separated. Specimens having an isotropic infill type with fibre orientation at zero degrees and one concentric ring showed maximum resistance to failure. It was also concluded that there is a correlation between filler material type and the pattern of the reinforcing material. Furthermore, it was observed that the performance of the parts can be improved by adding conferential rings, besides the conventional method of changing the angle of fibre only. Failure mode for all specimens was due to fibre pull-out. Before failure, nylon specimens reinforced with fibreglass and Kevlar were found to deflect more than carbon fibre with nylon.

Groo et al. [3] studied the estimation of structural performance due to fatigue damage and the life prediction of fibreglass composites which are commonly used in engineering structures such as wind turbine blades, automobiles, and aircraft, as they are subjected to dynamic loading with unpredictable operating conditions. It may be possible to significantly save maintenance costs and raise reliability if it is possible to monitor these structures while they are in use, and estimate how long they will last structurally. For in situ fatigue damage monitoring and lifespan prediction, this work used piezoresistive laser-induced graphene (LIG) integrated into fiberglass-reinforced composites. A transfer-printing method that is scalable and has the potential for automation was used to incorporate the LIG into fibreglass composites, lowering the obstacles to wider deployment.

In another study, Imeri et al. [4] evaluated the fatigue properties of fibre-reinforced specimens with a load ratio of 0.1 by changing the fibre pattern and infill type. Two types of fibre orientation patterns were selected, i.e., “concentric” and “isotropic”. From the experimental results, it was concluded that the greatest resistant to failure was that of carbon-fibre-reinforced nylon specimens with isotropic infill type and no rings. Further, it was observed that placing more concentric rings increased the fatigue life, which means that more cyclic loading will be needed for the failure to occur, while for isotropic infill, fatigue life decreases with the increase in the number of rings. In addition, it was concluded that there is a direct relation between the reinforcement material and the pattern type. Blais et al. [5] studied the fatigue testing of open hole samples fabricated using both printed holes and machined holes to assess the mechanical performance. Crucial factors affecting fatigue, such as material–property relationships and environmental conditions, require thorough exploration. Optimizing parameters like raster angle show promise, yet research on FDM fibre composites’ fatigue remains sparse. Addressing defects, considering viscoelasticity, and understanding the fibre properties’ impact are vital. Cellular geometry in materials like ACM significantly influences fatigue. Enhancing fatigue life necessitates a deeper exploration of these factors and their interplay to improve FDM polymeric materials and composites, particularly in biomedical and load-bearing applications. Shi Wang et al. [6] investigated the damage evaluation and failure of single-lap riveted joints and adhesive-riveted hybrid joints in carbon-fibre-reinforced plastic. The study revealed that even a slight increase in load results in a substantial decrease in the service life of riveted joints. The findings indicated that the fatigue life of CFRP riveted joints is highly sensitive to external excitation loads, with load increments causing a significant reduction in service life due to increased service temperatures. In this work, they discovered that the addition of adhesive significantly enhances the fatigue performance of riveted joints, particularly under high-load conditions, and mitigates the structure sensitivity to external excitation loads.

Jose et al. [7] investigated the mechanical properties such as the tensile strength, flexural strength, and impact energy of 3D-printed samples fabricated with FDM technology using PLA-graphene as raw material, by varying infill density and layer thickness. From the experimental results, it was observed that the mechanical properties improve as the linear layer thickness increases. In the case of the direct infill parameter, the behaviour was different. Tensile strength and flexural strength increase with the increase of infill, while the impact energy decreases as infill increases. Shanmugam et al. [8] studied the fatigue behaviour of 3D-printed polymer composites and it was noticed that the printing parameters, material characteristics, temperature, defects and voids, fibre properties, and cellular geometry and bulk material properties were crucial factors affecting the fatigue performance of FDM-based polymeric materials. The aim of this research was to evaluate the fatigue properties of FDM-printed polymeric materials, including biomedical applications and architected cellular materials. It also identified various factors that influence fatigue behaviour, including printing parameters, thermoplastic material characteristics, environmental temperatures, defects and voids, and fibre properties in composite materials. Bello et al. [9] investigated the effects of laser pre-treatment on improving the bonding of carbon-fibre-reinforced polymers (CFRPs) and increasing their resistance to mode-I fatigue crack growth. Uniform surface pre-treatment with a mid-infrared-range C_O2_ pulse laser was performed on the CFRP substrates, resulting in two surface morphologies—smooth, partially exposed fibres with low-energy irradiation and fully exposed fibres with high-energy irradiation laser ablation (LA). The results showed that uniform laser treatment increased the fatigue limit and improved the threshold surface energy release rate, GI_th_, compared to a baseline Teflon treatment. The laser ablation treatment achieved triple the strain energy release rate threshold value of the laser cleaning treatment. All treatments experienced adhesive failure, and plasticity in the adhesive was not observed. However, the crack growth rate sensitivity of the laser ablation treatment to the applied cycling load was high, limiting the time window for crack detection.

Pizzorni et al. [10] studied the effect of an adhesively bonded composite fabricated through the FDM technique and the failure behaviour of single-lap joints. A comprehensive experimental campaign was carried out to define how such a system responds to adhesive bonding after different surface treatments, using conventional pre-bonding preparations (degreasing or abrasion) and a more effective low-pressure plasma treatment. This allowed the determination of the joint performance and the identification of the current limits of the 3D-printed composite when its conditions are stressed in adhesive bonding. Mo Yang et al. [11] investigated the impact-induced damage and residual strength of carbon-fibre-reinforced polymer single-lap joints through a combination of finite element analysis and experimental methods. The results indicated a significant decrease in joint tensile strength after impact, with the residual strength of a 4 J impacted single-lap joint (SLJ) being 2029 N, compared to 4088 N for a non-impacted SLJ. In summary, this study demonstrated the effectiveness of the FEA model in predicting SLJ behaviour after transverse impact. It highlighted the impact energy threshold for significant damage and underscored the positive influence of increased overlap length on SLJ transverse impact resistance. Roy et al. [12] studied the tensile and compressive performance of adhesively bonded single-lap joints in green composites (bamboo fibre/Polylactic acid). The study investigated the impact of overlapping length and width on joint strength using epoxy, polyurethane, and PLA as bonding materials. The results indicated that increased width and overlapping length enhance tensile and compressive strength, with epoxy outperforming PLA and polyurethane. An analysis of variance identified width as crucial for tensile failure load and bonding material type as crucial for compressive failure load. The study recommended epoxy as the optimal bonding material for its stiffness and rigidity. The failure mode analysis revealed various failure types under different loading conditions.

In another study, Pizzorni et al. [13] worked on the improvement of the adhesive joint properties using 3D-printed parts which were reinforced using short and continuous carbon fibre. Several design factors such as overlap length, adhesive type, substrate thickness, and configuration were investigated to overcome their limitations. It was noted that the polyurethane performs better than epoxy following the joint deformation during testing; this prevents the substrate from delamination and reduces stress concentration. Garcia et al. [14] worked on the bond interface design for adhesively bonded single-lap joints fabricated using the additively manufacturing technique. Improvements in the shear strength of adhesively bonded lap joints were noticed for the samples fabricated by fusing structural reinforcements to the adherents through the FDM technique. Printed reinforcement appears to have imparted higher shear resistance to the bond regions. Khosavani et al. [15] worked on the structural integrity of adhesively bonded 3D-printed joints fabricated using the FDM method. The influence of process parameters and adhesive thickness on the performance of adhesively bonded joints was studied. An adhesive of 0.2 mm thickness was considered as the optimum value with highest mechanical strength. From the experiment, it was also clear that the main type of failure is cohesive. Jumpei et al. [16] proposed a method in their study for automatically embedding and strengthening CFRP in the thickness direction using lap joints, to address the issue of lower tensile strength in the thickness direction when using fused filament fabrication 3D printers. The study conducted tensile tests on CFRP-reinforced specimens with lap joints and found that strength loss can be suppressed by increasing the length of the lap joint and embedding a subsequent layer before curing the epoxy resin. The study also formulated the relationship between the lap joint length and modelling height and determined the minimum number of CFRPs of different lengths required for embedding, as well as exploring four different embedding methods. It was also suggested that the short CFRP, at 80% of the length of the long CFRP, maximizes the upper limit of the effective overlap joint length, and that a smaller effective overlap joint length reduces the overall layered joint length, leading to cost reduction.

The aim of this paper is to evaluate the fatigue performance of 3D-printed polymer composites reinforced with continuous carbon fibre with varying loading conditions. A lap shear test analysis for different bonding areas was also studied to see the effect of bonded samples versus the samples that were fabricated using 3D printing. In this study, the performance of adhesively manufactured specimens was evaluated against the samples fabricated as a single component, manufactured using the Markforged Mark Two 3D printer.

## 2. Materials and Methods

The Markforged Mark Two 3D printer was used in this study to fabricate the samples for testing. The Markforged Mark Two is a high-end 3D printer designed for industrial and engineering applications. It uses a unique printing process called “composite filament fabrication” (CFF) that allows it to print parts using a combination of different materials, including nylon, fiberglass, carbon fibre, and Kevlar. Continuous carbon fibre was used as a reinforcement material as it is strong, stiff, and lightweight, making it ideal for creating parts that require high strength and low weight. Nylon was used as the effective matrix filament and was supplied by Goprint3d, UK, a supplier of Markforged, Cambridge, MA, USA. It is the proprietary blend of Markforged, and has a diameter of 1.75 mm. The reinforcing carbon fibre (CF) was also supplied by Goprint3d, having a diameter of 0.35 mm impregnated with a sizing agent (nylon). The Markforged Mark Two is capable of printing parts with high strength and durability, making it ideal for applications where parts need to withstand high loads or stresses. It also features a large build volume and high accuracy, making it a versatile tool for prototyping and manufacturing.

**Figure 1:**
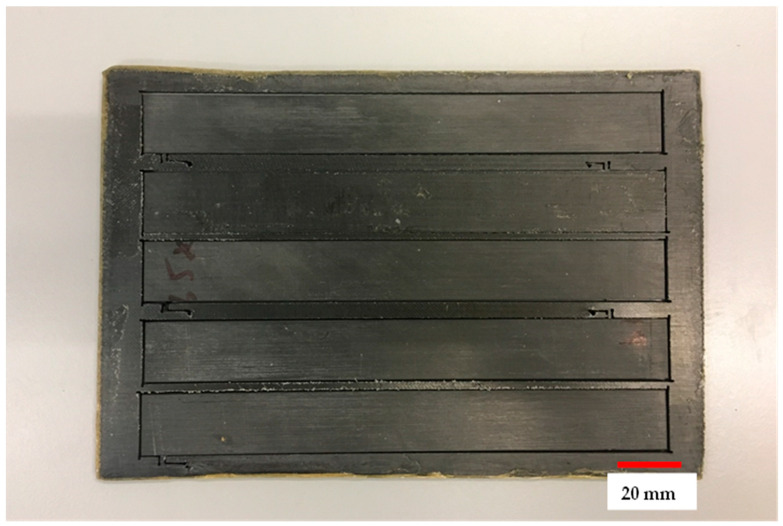
Sample after pressing using platen press.

To enhance the strength and modulus of the 3D-printed polymer composites, steps were taken to control the fibre volume fractions through two different methods. The first method involved adjusting the number of fibre layers in the composite specimens while maintaining controlled fibre orientation, with the specimens having the maximum number of fibre layers. The second technique utilized a hot platen press machine to increase the fibre volume fraction. By applying a pressure of 50 kPa at a temperature of 130 °C, the interface bonding between the fibre and matrix improved, and the voids induced during printing were reduced. Comparing the mechanical properties of the hot press samples (Figure 1) to the non-press samples, a significant enhancement was observed. The tensile strength and elastic modulus of the hot press samples approached those achieved through conventional composite manufacturing techniques. This technique offers a valuable means for researchers and designers to enhance the mechanical properties of 3D-printed polymer composites, particularly for applications requiring high-strength structures.

To use a Markforged Mark Two 3D printer, a 3D model of the part is created using 3D modelling software and then the 3D model is uploaded to the printer using Eiger (Markforged Mark Two slicing software); the material is then selected, and the printing process is started. The printer uses CFF technology to reinforce the printed part with continuous strands of fibre, resulting in a strong, lightweight, and durable part. Overall, the properties of continuous carbon fibre make it an ideal choice for high-performance applications that require strength, stiffness, and durability. However, it is also a relatively expensive material, which can limit its use in some applications. In this study, continuous carbon fibre was used as a reinforcement to fabricate polymer composites.

## 3. Test Conditions

**Figure 2:**
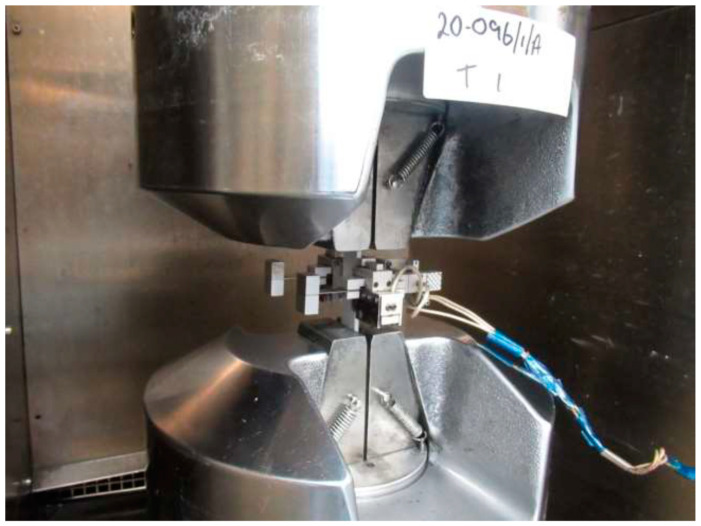
Test setup for the static test on polymer composite samples in the longitudinal direction.

This paper presents the results of fatigue tensile tests performed for the estimation of fatigue life in terms of the number of cycles to failure of the 3D-printed thermoplastic composites. Testing was carried out at ambient temperature on specimens that were unconditioned prior to testing. All tests were carried out in a laboratory environment controlled to 23 ± 2 °C and 50 ± 10% relative humidity. The specimens were manufactured using the Markforged Mark Two 3D printer according to the ASTM D3039 standard [17]. Samples were fabricated with 24 and 16 plies, with a layer thickness of 0.125 mm, deposited on the room temperature build platform. Continuous carbon fibre filament was reinforced with nylon to prepare 3D-printed samples which had a fibre volume content of 35%, as reported in the literature [18]. Static tests were conducted before fatigue analysis according to the parameters which are specified in the ASTM D3039 standard, as shown in Figure 2. A biaxial extensometer was used to measure strain in the longitudinal and transverse direction. A test speed of 2mm/min was used for all specimen types, and the elastic modulus was calculated from the stress strain curve in the elastic region. After this, fatigue testing was conducted at a frequency of 5 Hz with a load ratio of 0.1.

Fatigue occurs due to repeated cyclic loading which results in fluctuating stress. The load applied to the part might be less than the ultimate tensile strength, but the alternating nature of the load may break the part over time. On average, 50% to 90% failures occur due to fatigue loading. Some of the factors that affect the fatigue life are stress range, geometry, material type, and the environmental conditions. Fatigue failure occurs suddenly and is catastrophic, which makes it necessary to study for product development.

**Figure 3:**
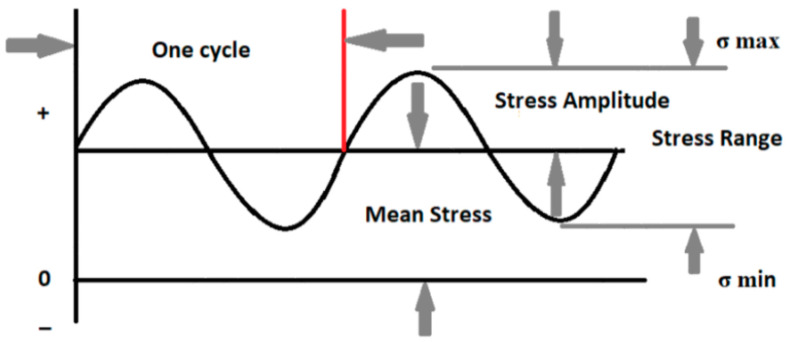
S-N curve terminologies for fatigue life estimation.

Before designing any part, some questions regarding part usage and applications need to be studied, such as the expected fatigue life of the part. If the part is designed for an infinite lifetime, the inspection intervals for the safe operation of the part must be understood. Fatigue life or the number of cycles to failure depends on several factors such as loading type, stress ratio, mean stress effects, and fatigue modification factors. Terminologies discussed in [19] that are mainly used in assessing fatigue life are shown in Figure 3.

$$\mathrm{Stress}\;\mathrm{ratio},\;R=\frac{\sigma_{min}}{\sigma_{max}},$$

where $\sigma_{min}$ is the minimum stress and $\sigma_{max}$ is the maximum stress.

$$\mathrm{Stress}\;\mathrm{range},\;\Delta \sigma =\sigma_{max}-\sigma_{min}$$

$$\mathrm{Mean}\;\mathrm{stress},\;\sigma_{m}=\frac{\sigma_{max}+\sigma_{min}}{2}$$

Fully reversed loading occurs when an equal and opposite load is applied in which $\sigma_{m}$ is zero and R = −1, while zero-based loading occurs when a load is applied and removed in which $\sigma_{m}$ is $\frac{\sigma_{max}}{2}$ and R = 0.

Fatigue life assessment is critical in designing the structure of a component used in the aerospace, medical, marine, and automobile industries. It gives the designer an estimate of the component service life with safe operation. The fatigue behaviour of 3D-printed polymer composites has not been reviewed in detail; therefore, this study aims to evaluate the performance of fatigue life of samples fabricated using the FDM technique. Fatigue tests were carried out on 3D-printed polymer composites using different loading conditions with fibre orientation in a unidirectional orientation. Static tests were carried out to determine the ultimate tensile strength using tensile testing, and from those data, the load was varied from 80 to 98% of the total static load for fatigue testing.

## 4. Lap Shear Testing (Analysis)

Lap shear strength (LSS) testing involves the axial pulling of the bonded specimen, and it is one of the most commonly used test methods for investigating bond strength. The following equation can be used to calculate LSS.

$$\tau =\frac{F_{max}}{Lxb}\;N/{\mathrm{mm}}^{2}$$

where τ is the lap shear strength, *L* is the length of the overlap, *b* is the width of the overlap area, and *F_max_* is the maximum tensile force.

**Figure 4:**
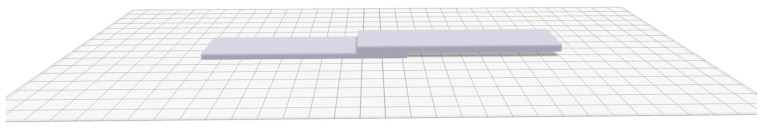
Lap shear sample in Eiger slicing software as a single part.

**Figure 5:**
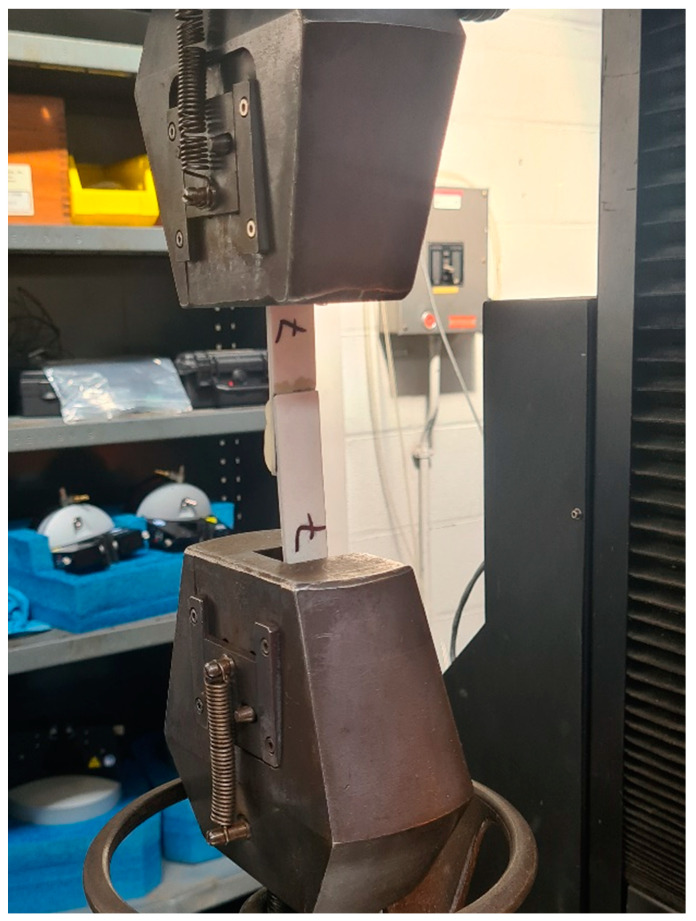
Lap shear sample during testing.

Lap shear testing was carried out according to the ASTM D5868 standard [20] as designed in a single piece shown in Figure 4. At least five samples of each type were tested to determine the lap shear strength of the 3D-printed parts bonded with Araldite adhesive and the samples that were prepared directly using 3D printing techniques. The dimensions of the samples were measured before each test, and the tests were performed for unidirectional samples. Figure 5 shows the sample mounted on the tensile machine during testing.

**Figure 6:**
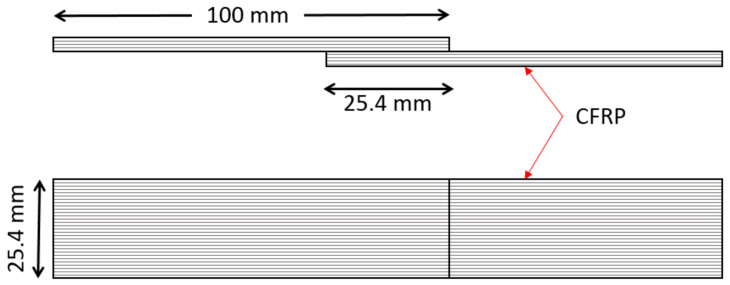
Lap shear sample dimensions.

Standard tensile tests were performed on 3D-printed adhesively bonded samples and 3D-printed samples as a single piece (Figure 6) using a hydraulic tensile test machine with a load cell of 10 kN. Aluminium tabs were bonded on both sides of the single-lap shear sample using Araldite adhesive to make sure that the applied load was in the plane of the adhesive layer during the tensile test. In this study, five samples for each test type were tested with repeatable results. Different types of failure were observed in lap shear samples which include (a) adhesive failure, (b) failure near the grips area, and (c) failure in adjacent structures. Maximum failure load was obtained for adhesive failure samples with a 2000 N load, followed by the cohesive failure sample with a load of 1500 N.

**Figure 7:**
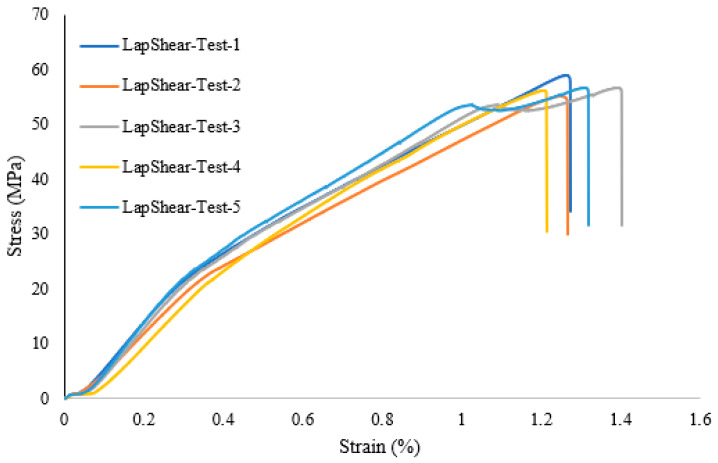
Lap shear strength of 3D-printed samples bonded using adhesive (25 mm width).

**Figure 8:**
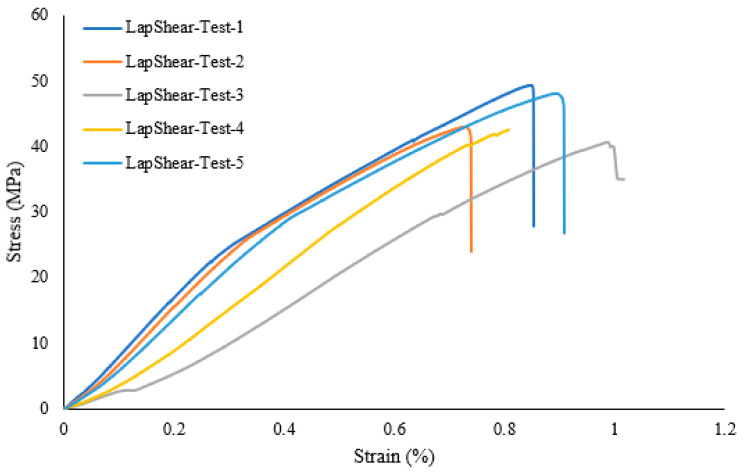
Lap shear strength of 3D-printed samples bonded using adhesive with a width of 12 mm.

**Figure 9:**
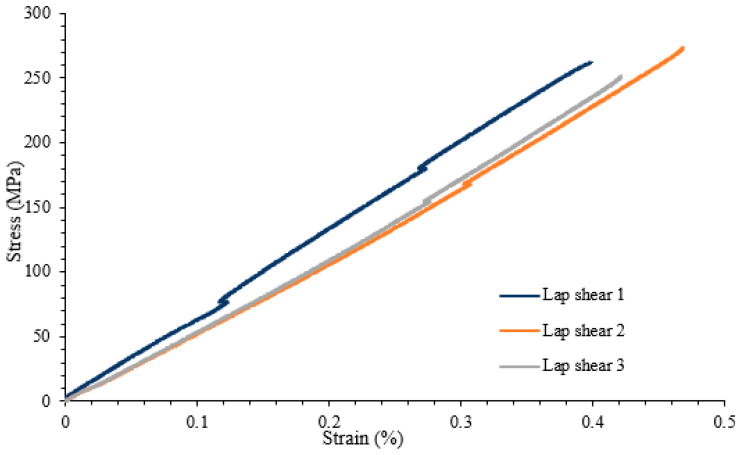
Lap shear strength of 3D-printed samples bonded as a single piece with a width of 12 mm.

A lap shear analysis of the 3D-printed samples was carried out on both the samples that were bonded using glue and the samples that were fabricated as a single piece using the Markforged Mark Two 3D printer. From the experimental results, it is clear that the single-piece sample exhibits higher strength as compared to the bonded samples using adhesive. Figure 7 shows the lap shear strength of adhesively bonded samples with an average strength of 56.47 MPa, while Figure 8 shows the lap strength of samples with 12 mm of bonded area, and from both these graphs, it is clear that the lap shear strength mainly depends on the bonded area. Figure 9 shows the lap shear strength of 3D-printed samples produced as a single piece with a width of 12 mm.

## 5. Results and Discussion

**Figure 10:**
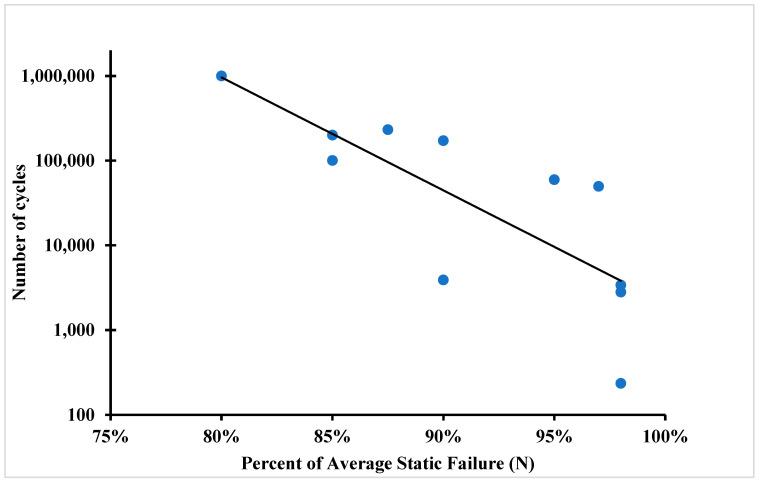
S-N curve of 3D-printed polymer composites for 24 layers of carbon fibre polymer composite.

**Figure 11:**
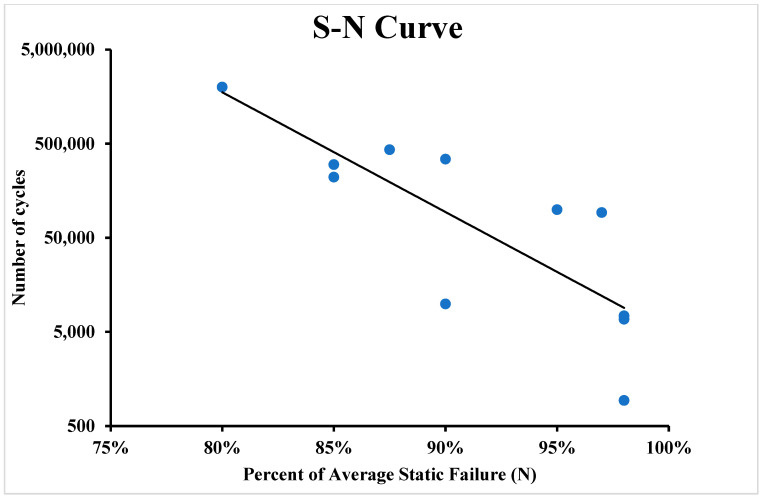
S-N curve of 3D-printed polymer composites for 16 layers of carbon fibre polymer composite.

During the experiment, all samples were successfully tested and separated in the shear area. The failure in the shear area means that the average tensile strength was not exceeded during the test. The fatigue limit or the S-N curve (also known as the fatigue curve or Wöhler curve) is frequently used to explain the fatigue properties of materials. The relationship between cyclic stress amplitude and the number of cycles to failure is illustrated by the S-N curve in Figure 10 and Figure 11. The number of cycles before failure is displayed using a logarithmic scale. The stress amplitude of the cyclic loading is the highest stress, which can be either linear or logarithmic.

Table 1 show the number of cycles to failure for the fatigue samples until failure at 80% of UTS. It took one million cycles for the samples, but failure did not occur. After that, samples were tested at 85% of the UTS, and from Table 2, it can be seen that the average number of cycles until failure was about 192,566 cycles, and the failure types was the splitting of the samples.

Table 3 shows the fatigue test carried out at 90% of the UTS, and it can be seen that the average number of cycles for the failure was about 51,738 cycles and the failure for this kind of samples was splitting and failure near the gripping region (clamp failure). Table 4 shows the fatigue results for the samples at 98% of the UTS; the number of cycles until failure were 2921 cycles, and the failure mode was splitting and clamp failure. For the sample with higher fibre volume fraction due to post-print pressing, fatigue life was higher compared to that observed by Giannakis et al. [21], as most of the load is taken by the fibre in the composite sample.

## 6. Dynamic Mechanical Analysis (DMA)

Dynamic mechanical analysis was carried out using Q 800 (TA instruments, New Castle, DE, USA) test setup, with sample dimensions of 55 mm × 12.73 mm × 3.46 mm and a span length of 30 mm, using ambient temperature to determine the loss and storage modulus of the polymer composites. The specimens were exposed to a temperature sweep from 30 °C to 120 °C at a constant 1 °C/min rate and 1 Hz frequency. DMA is a technique used to study the mechanical behaviour of materials as a function of temperature, frequency, and time. Polymer composites reinforced with continuous carbon fibre (CCF) are an excellent candidate for DMA analysis due to their unique mechanical properties.

**Figure 12:**
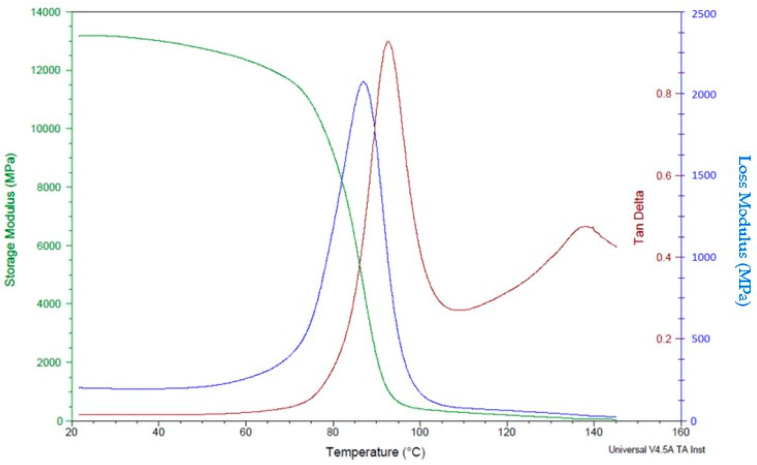
DMA analysis of polymer composite samples with 24 fibre layers.

**Figure 13:**
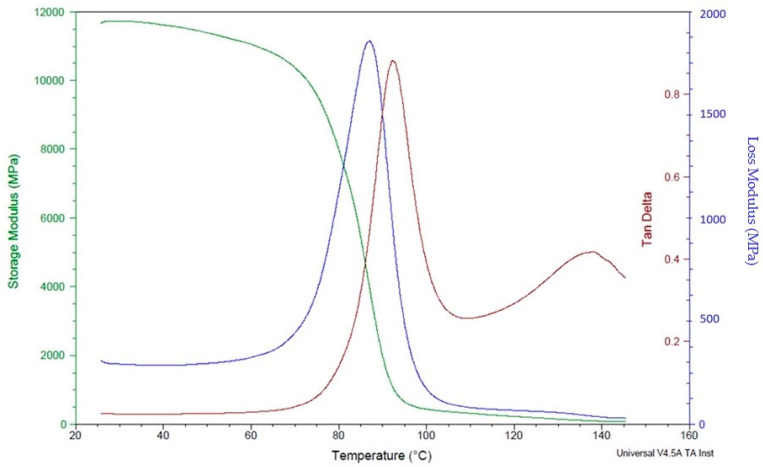
DMA analysis of polymer composite samples with 16 fibre layers.

The DMA analysis of polymer composites reinforced with CCF can provide valuable information about the material’s stiffness, damping, and viscoelastic behaviour. Overall, the DMA analysis of polymer composites reinforced with CCF can provide valuable information about the material’s mechanical behaviour, which is essential for designing and optimizing high-performance components for applications in industries such as aerospace, automotive, and defence. The dynamic mechanical analysis test reveals higher storage and loss modulus in specimens with a high number of carbon layers (Figure 12) compared to specimens with a lower number of fibre content (Figure 13).This is mainly attributed to the better mechanical restraints imposed by carbon fibres on the matrix movement.

In this study, DMA was used to examine how the amount of fibre affects the mechanical behaviour of composites made of nylon and carbon fibre that are fabricated using the Markforged Mark Two 3D printer. Since the molecules in the matrix material are disturbed and there is more room for molecular motion at higher temperatures, the viscoelastic behaviour is accelerated [22]. The polymeric matrix loses mechanical rigidity and creep resistance as it gets softer and eventually turns rubbery. In a previous study, Fernandes et al. [23] assessed the mechanical properties of thermoplastic reinforced 3D composites with different infill types. Experimentally determined values were compared with finite element analysis (FEA) based on classical composite failure criteria. DMA was used to characterise the viscoelastic properties of continuous fibre composites produced additively. The findings demonstrated that continuous fibres raised the glass transition temperature by 53.8% (concentric) and 44.6% (isotropic), respectively, indicating that molecules need more energy to reach the rubbery state.

Ibrahim et al. [24] studied the dynamic mechanical analysis and mechanical behaviour of 3D-printed polymer composites reinforced with nylon using three build orientations: angular, vertical, and horizontal. Dynamic mechanical analysis, flexural tests, and tensile tests were conducted to assess the specimens. It was noted from the results of the dynamic mechanical analysis test that the horizontal specimens have larger storage and loss moduli, enhanced damping capabilities, and improved flexural and tensile properties compared to the vertical and angular specimens. This is mostly attributable to the superior mechanical constraints that carbon fibres place on the movement of the matrix. Scanning electron microscopy revealed that the alignment of carbon fibres in the direction of the applied load contributes to the higher stiffness of the horizontally printed samples.

## 7. Fourier Transform Infrared (FTIR) Analysis

FTIR analysis is a type of spectroscopy that involves measuring the absorption or transmission of infrared radiation by a sample. This technique is commonly used to identify and quantify the chemical bonds present in a sample and can provide information about the functional groups and chemical structure of the molecules in the sample. In FTIR analysis, a beam of infrared radiation is passed through a sample, and the resulting spectrum is recorded. The spectrum shows how much of the radiation is absorbed by the sample at different wavelengths and can be used to identify the types of chemical bonds present in the sample. The data can be analysed using software to determine the identity and quantity of the components in the sample.

**Figure 14:**
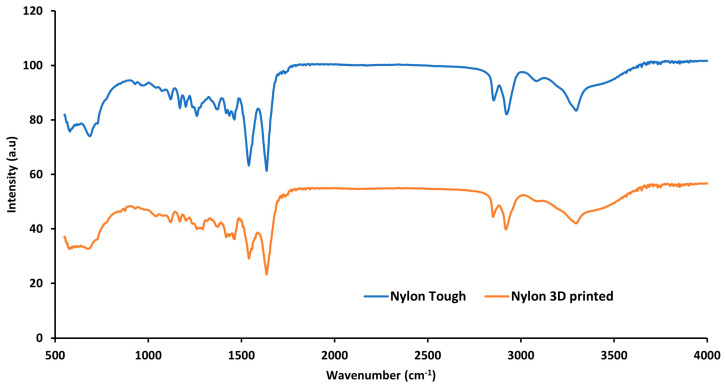
FTIR analysis of tough nylon and 3D-printed polymer composite.

In Figure 14, the FTIR spectra of nylon sample are presented. The figure demonstrates all the major bands associated with nylon samples. The figure reveals medium band peaks at 3298 cm^−1^ and 2934 cm^−1^ that can be attributed to the N-H stretch from amino groups and the C-H stretching vibration from alkane groups, respectively. The strong peaks at 1636 cm^−1^ and 1535 cm^−1^ and all other peaks in the range of 1500 cm^−1^ to 1700 cm^−1^ are due to amide I and II bands. The peak at 1535 cm^−1^ is associated with the amide II band which appeared due to C-N stretching and N-H bonding. The peak at 1636 cm^−1^ is associated with C=O stretching from the carbonyl group. No significant difference was found between the nylon material before and after processing (3D printing).

## 8. Conclusions

From the experiments that were performed on the 3D-printed samples, it was concluded that the fibre volume fraction has a great influence on the fatigue life of the additively manufactured polymer composite samples. Fibre volume fraction affects the fatigue properties; it was concluded that the higher the volume fraction, the better the fatigue life of the polymer composites. In addition, testing was performed on samples near the endurance limit to determine the exact point on the S-N curve. This study revealed that the fibre properties and loading percentage are crucial factors that affect the determination of fatigue life. The specimens exhibited various fracture types such as splitting, delamination, and fracture at the clamp region. For the samples tested with 80% of the ultimate tensile strength, fracture did not occur even after one million cycles, while samples tested with 98% of the ultimate tensile strength experienced failure after 2900 cycles. This study concluded that samples with a volume fraction of 30% had higher fatigue strength than those with a volume fraction of 20%. Moreover, in lap shear testing, it was observed that samples printed as a single part had a higher tensile strength (265 MPa) than those that were adhesively bonded (56 MPa) with a 12 mm and 25 mm adhesively bonded area.

## Figures and Tables

**Table 1:** Fatigue test results at a stress ratio of 0.1 with 5 Hz frequency at 80% of UTS.

Specimen Number	Thickness (mm)	Width (mm)	Max Load (kN)	Min Load (kN)	Load Percent (%)	No. of Cycles	Failure Modes
1	3.50	19.03	27.088	2.709	80%	1,000,000	None
2	3.41	19.0	27.088	2.709	80%	1,000,000	None
3	3.43	18.96	27.088	2.709	80%	1,000,000	None

**Table 2:** Fatigue test results at a stress ratio of 0.1 with 5 Hz frequency at 85% of UTS.

Specimen Number	Thickness (mm)	Width (mm)	Max Load (kN)	Min Load (kN)	Load Percent (%)	No. of Cycles	Failure Modes
1	3.46	19.06	28.781	2.878	85%	200,036	Splitting
2	3.46	19.05	28.781	2.878	85%	177,175	Splitting
3	3.48	19.01	28.781	2.878	85%	200,488	Splitting

**Table 3:** Fatigue test results at a stress ratio of 0.1 with 5 Hz frequency at 90% of UTS.

Specimen Number	Thickness (mm)	Width (mm)	Max Load (kN)	Min Load (kN)	Load Percent (%)	No. of Cycles	Failure Modes
1	3.46	18.99	30.474	3.047	90%	59,538	Clamp failure
2	3.43	18.96	30.474	3.047	90%	49,771	Clamp failure
3	3.39	18.97	30.474	3.047	90%	45,906	Splitting

**Table 4:** Fatigue test results at a stress ratio of 0.1 with 5 Hz frequency at 98% of UTS.

Specimen Number	Thickness (mm)	Width (mm)	Max Load (kN)	Min Load (kN)	Load Percent (%)	No. of Cycles	Failure Modes
1	3.48	19.06	33.183	3.318	98%	3400	Splitting
2	3.46	19.09	33.183	3.318	98%	2813	Splitting
3	3.46	19.06	33.183	3.318	98%	2552	Clamp failure

## Data Availability

Data are contained within the article.
